# The architecture of quality of life after stroke: a network comparison of mild-to-moderate versus severe functional impairment

**DOI:** 10.3389/fneur.2026.1808859

**Published:** 2026-08-19

**Authors:** Jingjing Ma, Yiqing Zhang, Tao Zhou, Lulu Tong, Lu Shi

**Affiliations:** 1 Department of Nursing, Ningbo Medical Center Lihuili Hospital, Ningbo, Zhejiang, China; 2 Rehabilitation Ward, Ningci Rehabilitation Hospital, Ningbo, Zhejiang, China; 3 Department of Rehabilitation Medicine, Zhejiang Provincial People’s Hospital, Hangzhou, Zhejiang, China; 4 Department of Rehabilitation, Ningbo Medical Center Lihuili Hospital, Ningbo, Zhejiang, China

**Keywords:** activities of daily living (ADL), network analysis, quality of life, rehabilitation, self-care, stroke

## Abstract

**Background:**

Health-related quality of life (HRQoL) after stroke is multidimensional, yet conventional analyses often overlook the complex interactions among its domains and how these might differ by functional status.

**Objective:**

This study aimed to model and compare the network architecture of HRQoL between stroke survivors with mild-to-moderate versus severe functional dependence.

**Methods:**

In this multi-center cross-sectional study, 451 inpatients were assessed using the Stroke-Specific Quality of Life (SS-QOL) scale across 12 domains. Participants were stratified into a Mild–Moderate group (Barthel Index, BI > 40; *n* = 259) and a Severe group (BI ≤ 40; *n* = 192). Polychoric correlations were used to account for the ordinal nature of the SS-QOL items. Strength centrality and bridge expected influence (based on *a priori* communities: Physical, Psychological, Social, General) were calculated to identify hubs and bridges. Network stability was assessed via bootstrap analysis, and a Network Comparison Test (NCT) was performed to compare global strength and network structure between groups.

**Results:**

Although network structure did not differ significantly between groups (*p* = 0.928), the severe dependence group showed significantly higher global strength (*p* = 0.021), suggesting a more densely connected HRQoL network. In the Mild–Moderate group, strength centrality was highest for Self-Care (S7; 1.209) and Social Roles (S8; 1.014). In the Severe group, strength centrality was highest for Upper Extremity Function (S10; 1.124) and Thinking (S9; 1.031). Bridge expected influence was highest for Thinking (S9; 0.694) in the mild–moderate group and for Language (S3; 0.722) and Thinking (S9; 0.701) in the severe group. Network stability was acceptable in both groups (CS-coefficients: 0.595 and 0.438 for strength centrality).

**Conclusion:**

The fundamental architecture of post-stroke HRQoL differs across levels of functional dependence. These findings support a precision rehabilitation framework: one targeting the synergy between physical functions in milder cases, and another focused on supporting cognitive integrity and social adaptation in severe disability.

## Introduction

Stroke is a leading global cause of long-term disability. With approximately 12 million new cases annually and an estimated 94 million survivors living with its sequelae (1), the burden is immense and growing. China shoulders a significant portion of this burden, with a nationwide surveillance programme reporting 3.4 million incident stroke cases among adults aged ≥40 years in 2020 alone (2). Post-stroke disability is highly prevalent, affecting an estimated 70%–80% of Chinese survivors with varying degrees of motor and functional impairment (3). These deficits not only compromise neurological function and independence but also exert a profound and enduring negative impact on health-related quality of life (HRQoL) across physical, psychological, and social domains (4, 5). Consequently, improving HRQoL has emerged as a paramount objective in contemporary stroke rehabilitation science.

Health-related quality of life (HRQoL) in the context of stroke is a patient-centered, multidimensional construct. World Health Organization’s (WHO) classic definition of quality of life: “individuals perceptions of their position in life in the context of the culture and value systems in which they live and in relation to their goals, expectations, standards and concerns” (6). For stroke survivors, this subjective experience is systematically captured by instruments such as the Stroke-Specific Quality of Life (SS-QOL) scale (7). The SS-QOL operationalizes HRQoL into distinct yet interrelated domains including energy, mood, thinking, self-care, social roles, family roles, and upper extremity function, among others. Consequently, post-stroke HRQoL represents an integrated state shaped by the dynamic interplay of physical capability, psychological well-being, and social participation.

However, conventional approaches to assessing post-stroke HRQoL face methodological constraints. Firstly, most studies rely on aggregate summary scores or linear regression models. While useful for identifying independent predictors, these methods inherently treat HRQoL domains as separate components, failing to capture their interdependent relationships and potential causal dynamics within a holistic system. This limitation is highlighted by recent critiques noting that the complex interdependences of post-stroke outcomes cannot be sufficiently depicted by commonly used uni- or bivariate analyses (8). Secondly, by treating all stroke survivors as a single, homogeneous group, existing research may obscure the potentially distinct challenges and mechanisms that underlie HRQoL for patients with different levels of functional dependence. Emerging network analytic evidence strongly supports this concern by demonstrating that the network structure of influencing factors differs between patient groups stratified by functional outcome (9). Consequently, a critical gap exists in our understanding of how the very structure of life quality varies across the spectrum of stroke severity, which is essential for developing stratified and personalized rehabilitation strategies.

To address these limitations, psychological network analysis offers a useful tool. This method models HRQoL as an interactive network, where domains (e.g., Mobility, Mood) are connected. Network analysis can map associations among domains and identify which ones are most central (10). Second, this approach allows us to create and compare separate network maps for different groups of patients. This direct comparison can test whether the structure of post-stroke HRQoL differs depending on a patient’s level of disability, a key step for personalizing rehabilitation strategies (11).

The ICF model describes an individual’s lived experience of health as an outcome of dynamic interactions between their health condition (e.g., stroke), body functions and structures (e.g., motor skills, cognition), activities (e.g., self-care), participation (e.g., social roles), and contextual environmental and personal factors (12). The weight and pattern of these interactions are not fixed but may vary systematically across individuals with different levels of impairment. Therefore, investigating whether the network structure of HRQoL (which directly corresponds to ICF constructs) differs between patients with mild-to-moderate versus severe functional dependence constitutes a direct empirical test of this core theoretical premise. This approach aligns with recent quantitative research that utilizes the ICF framework in stroke populations (13).

Grounded in the ICF theoretical framework and utilizing network analysis, the study aimed to model and compare the network architecture of health-related quality of life (HRQoL) in stroke survivors stratified by functional dependence: mild-to-moderate (Barthel Index, BI > 40) versus severe (BI ≤ 40). Our primary objectives were twofold: (1) to estimate and compare the network structures of HRQoL domains separately for patients with mild-to-moderate and severe functional impairment; and (2) to identify the most central domains and bridge nodes within each group, as well as differences in network properties (global strength, structure, and individual edges) between the two groups. We sought to provide an empirical understanding of how the structure of life quality varies across the spectrum of stroke severity.

## Methods

### Study design and setting

This was a multi-center, cross-sectional study. Data were collected from the rehabilitation departments of three hospitals in Zhejiang Province, China, between October 20, 2024, and December 1, 2025. The sites comprised two public tertiary hospitals (one provincial and one municipal), covering both urban and rural areas, and one private rehabilitation hospital.

### Participants

Stroke survivors were recruited via convenience sampling across all three centers. The final analytical sample included 451 inpatients.

#### Inclusion criteria were

(1) Diagnosis of stroke according to the Chinese Diagnostic Criteria for Major Cerebrovascular Diseases (2019); (2) Aged ≥18 years; (3) Medically stable, not in intensive care, and requiring no urgent intervention.

#### Exclusion criteria included

Diagnosis of a severe systemic disease, altered consciousness during the study period, inability to provide reliable self-report due to severe aphasia or cognitive impairment, or withdrawal of informed consent. All participants provided voluntary, written informed consent. The study protocol was approved by the appropriate Institutional Review Boards.

#### Sample size justification

Network analysis was performed on 451 patients. While estimation guidelines suggest simpler networks can be reliably estimated with approximately 250 observations, more complex networks often require larger samples of about 500 for stability. Our sample of 451 lies between these benchmarks and was deemed sufficient. Subsequent stability analysis confirmed the reliability of estimates. The correlation stability (CS) coefficient for strength centrality was 0.595 for the mild–moderate group (good) and 0.438 for the severe group (acceptable), both exceeding the recommended threshold of 0.25 (14).

### Measures and data collection

Data were collected via structured questionnaires, supplemented by information extracted from medical records. All data collectors received standardized training to ensure consistency.

### Demographic and clinical characteristics

Demographic data for patients included age, sex, marital status, education, occupation, monthly household income, and primary payment method for medical costs. Clinical data included stroke severity, assessed using the National Institutes of Health Stroke Scale (NIHSS), and functional dependence level, classified based on Activities of Daily Living (ADL) scores obtained from medical records. The cutoff of BI = 40 was selected to define severe functional dependence, as this threshold has been widely used in stroke research to distinguish severe from mild-to-moderate impairment.

### Stroke-specific quality of life (SS-QOL)

Health-related quality of life was assessed using the Chinese version of the Stroke-Specific Quality of Life Scale (SS-QOL) (15). This scale contains 49 items across 12 domains: Energy, Family Roles, Language, Mobility, Mood, Personality, Self-Care, Social Roles, Thinking, Upper Extremity Function, Vision, and Work/Productivity, plus one item on general self-rated health. Items are rated on a 5-point Likert scale (e.g., 1 = “could not do it at all” to 5 = “no trouble at all”), with higher scores indicating better QoL. According to the standard SS-QOL scoring protocol, negatively worded items were reverse-scored prior to domain score calculation. The scale has excellent overall reliability (Cronbach’s *α* = 0.98), and domain-level internal consistency ranges from 0.77 to 0.95 as reported in the literature (16). The scores from these 12 domains served as the primary nodes for the network analysis. The total SS-QOL score was used as a secondary outcome for overall sample description.

### Statistical analysis

Descriptive analyses were performed using SPSS 25.0. Continuous variables are presented as mean ± standard deviation or median with interquartile range, as appropriate. Categorical variables are summarized as frequencies and percentages.

Network estimation. The core network analysis was implemented in R (version 4.4.2) using the bootnet and qgraph packages. Given the ordinal nature of the SS-QOL items (Likert scales), we used polychoric correlations for all network estimations, implemented via the cor_auto function. A partial correlation network among the 12 SS-QOL domains was estimated using the EBICglasso method with the Extended Bayesian Information Criterion (EBIC) for model selection (gamma = 0.5), producing a sparse network of conditional dependence relationships. In the resulting graph, nodes represent QoL domains, and edges represent regularized partial correlations. Blue edges depict positive associations, while red dashed edges depict negative associations; edge thickness corresponds to the absolute strength of the association.

Strength centrality was calculated as the sum of absolute edge weights connected to a node. Bridge expected influence (1-step) was calculated as the sum of edge weights from a node to nodes belonging to different communities. Communities were defined *a priori* based on the ICF framework: Physical (Mobility, SelfCare, Upper Extremity Function, Vision), Psychological (Mood, Personality, Thinking), Social (Family Roles, Social Roles, Productivity), and General (Energy, Language). Predictability (R^2^) was calculated as the proportion of variance in each node explained by its neighbors. Betweenness and closeness were not interpreted due to their known instability in psychological networks (14). The stability of edge weights and centrality indices was assessed using a case-dropping bootstrap procedure (1,000 samples). The correlation stability (CS) coefficient was used to quantify robustness, with values > 0.50 considered good and > 0.25 considered acceptable. Network differences between the two groups were formally tested using the Network Comparison Test (NCT) with 1,000 bootstrap iterations, examining global strength, network structure (M-test), and individual edges.

### Sensitivity analyses

We performed several sensitivity analyses to assess robustness. A Network Comparison Test (NCT) was conducted to compare global strength and network structure between the two groups (Supplementary Table S1). Additional sensitivity analyses included Spearman correlations (Supplementary Table S2), alternative community partition (Supplementary Table S3), residualized networks (Supplementary Table S4), bootstrap verification of the negative edge (Supplementary Table S5), an alternative BI cutoff (Supplementary Table S6), node predictability (R^2^; Supplementary Table S7), and removal of BI-overlapping domains (Supplementary Table S8). Results remained generally consistent across all analyses, supporting the robustness of our findings.

## Results

### Sample characteristics

Initial screening identified 500 eligible stroke survivors. Subsequently, 49 participants were excluded due to the following reasons: participant refusal (*n* = 20), failure to meet inclusion criteria (*n* = 15), acute clinical deterioration (*n* = 3), loss to follow-up (*n* = 2), withdrawal by patient (*n* = 2), or submission of essentially incomplete data (*n* = 7). The final analytical sample comprised 451 participants. Among the 451 included participants, complete data were available for all 12 SS-QOL domains and the Barthel Index. No missing values were present in the variables used for network analysis.

The final analytical sample comprised 451 participants, stratified by their Barthel Index (BI) scores into two groups: those with BI > 40 (Mild–Moderate ADL Impairment Group, *n* = 259, 57.4%) and those with BI ≤ 40 (Severe ADL Impairment Group, *n* = 192, 42.6%). As detailed in Table 1, the Severe ADL Impairment Group was significantly older, had lower educational attainment and monthly income, and had more severe clinical profiles including a higher proportion of hemorrhagic stroke and greater neurological impairment on the NIHSS, compared to the Mild–Moderate ADL Impairment Group. Most notably, quality of life was substantially lower in the Severe ADL Impairment Group (median = 2.22, Q_1_−Q_3_: 1.65–2.96) than in the Mild–Moderate ADL Impairment Group (median = 3.73, Q_1_−Q_3_: 3.13–4.26; Z = −13.97, *p* < 0.001) (Table 1).

**Table 1:** Demographic and clinical characteristics of stroke survivors stratified by activities of daily living (Barthel index).

Characteristic	Total (*n* = 451)	Mild–moderate ADL impairment group, BI > 40 (*n* = 259)	Severe ADL impairment group, BI ≤ 40 (*n* = 192)	Statistic	*p*
Gender, *n* (%)				*χ*^2^ = 0.04	0.839
Male	296 (65.63)	171 (66.02)	125 (65.10)		
Female	155 (34.37)	88 (33.98)	67 (34.90)		
Age, *n* (%)				*χ*^2^ = 14.80	0.002
<45	45 (9.98)	32 (12.36)	13 (6.77)		
45–59	107 (23.73)	63 (24.32)	44 (22.92)		
60–75	172 (38.14)	108 (41.70)	64 (33.33)		
>75	127 (28.16)	56 (21.62)	71 (36.98)		
Marital status, *n* (%)				*χ*^2^ = 7.24	0.065
Married	363 (80.49)	213 (82.24)	150 (78.12)		
Single	18 (3.99)	10 (3.86)	8 (4.17)		
Widowed	48 (10.64)	20 (7.72)	28 (14.58)		
Divorced/Other	22 (4.88)	16 (6.18)	6 (3.12)		
Education level, *n* (%)				*χ*^2^ = 7.96	0.047
Primary or below	199 (44.12)	101 (39.00)	98 (51.04)		
Middle school	124 (27.49)	73 (28.19)	51 (26.56)		
High school	69 (15.30)	46 (17.76)	23 (11.98)		
College/bachelor’s or above	59 (13.08)	39 (15.06)	20 (10.42)		
Employment, *n* (%)				*χ*^2^ = 7.38	0.061
Employed	95 (21.06)	65 (25.10)	30 (15.62)		
Retired	226 (50.11)	128 (49.42)	98 (51.04)		
Unemployed	58 (12.86)	28 (10.81)	30 (15.62)		
Other	72 (15.96)	38 (14.67)	34 (17.71)		
Medical payment, *n* (%)				*χ*^2^ = 0.66	0.717
Rural medical insurance	102 (22.62)	55 (21.24)	47 (24.48)		
Urban medical insurance	318 (70.51)	186 (71.81)	132 (68.75)		
Out-of-pocket/Commercial	31 (6.87)	18 (6.95)	13 (6.77)		
Monthly income, *n* (%)				*χ*^2^ = 7.76	0.021
<3,000 CNY	225 (49.89)	115 (44.40)	110 (57.29)		
3,000–5,000 CNY	187 (41.46)	121 (46.72)	66 (34.38)		
≥5,000 CNY	39 (8.65)	23 (8.88)	16 (8.33)		
Stroke type				*χ*^2^ = 14.85	0.002
Ischemic	241 (53.44)	151 (58.30)	90 (46.88)		
Hemorrhagic	100 (22.17)	41 (15.83)	59 (30.73)		
Other	86 (19.07)	54 (20.85)	32 (16.67)		
Mixed	24 (5.32)	13 (5.02)	11 (5.73)		
Comorbidities, *n* (%)					
Hypertension	281 (62.31)	155 (59.85)	126 (65.63)	*χ*^2^ = 1.57	0.210
Diabetes	131 (29.05)	74 (28.57)	57 (29.69)	*χ*^2^ = 0.07	0.796
Hyperlipidemia	64 (14.19)	37 (14.29)	27 (14.06)	*χ*^2^ = 0.00	0.946
Coronary disease	35 (7.76)	19 (7.34)	16 (8.33)	*χ*^2^ = 0.15	0.695
NIHSS score, *n* (%)				*χ*^2^ = 147.22	<0.001
Minor impairment (1–4)	225 (49.89)	189 (72.97)	36 (18.75)		
Moderate impairment (5–15)	174 (38.58)	67 (25.87)	107 (55.73)		
Moderately severe impairment (>15)	52 (11.53)	3 (1.16)	49 (25.52)		
Time post-stroke, *n* (%)				*χ*^2^ = 5.76	0.056
Acute (≤3 months)	398 (88.25)	236 (91.12)	162 (84.38)		
Subacute (4–6 months)	20 (4.43)	7 (2.70)	13 (6.77)		
Chronic (>6 months)	33 (7.32)	16 (6.18)	17 (8.85)		
Quality of life, median (Q₁, Q₃)	3.16 (2.36, 3.95)	3.73 (3.13, 4.26)	2.22 (1.65, 2.96)	Z = −13.97	<0.001

BI = Barthel Index; NIHSS = National Institutes of Health Stroke Scale; Q₁ = first quartile; Q₃ = third quartile. Categorical variables are presented as n (%), compared using the Chi-square test. The continuous variable (Quality of Life) is presented as median (Q₁, Q₃), compared using the Mann–Whitney U test (Z statistic).

### Distribution of scores across quality of life domains

Table 2 presents the median scores of the twelve quality of life (QoL) dimensions for both patient groups. Across all dimensions, the Severe ADL Impairment Group reported consistently and substantially lower median scores compared to the Mild–Moderate ADL Impairment Group. The most pronounced disparities were observed in Mobility (S4) (Severe: 1.50 vs. Mild–Moderate: 4.00), Self-care (S7) (1.70 vs. 4.00), and Productivity (S12) (1.00 vs. 3.00). Upper Extremity Function (S10) (1.70 vs. 4.00), Social Roles (S8) (1.60 vs. 3.40), and Thinking (S9) (2.00 vs. 4.00) also showed marked reductions. In contrast, Vision (S11) exhibited the smallest relative difference between groups (Severe: 3.67 vs. Mild–Moderate: 5.00) (Table 2).

**Table 2:** Median scores of quality of life dimensions in stroke patients.

Dimension	Mild–moderate ADL impairment group, BI > 40 (*n* = 259)		Severe ADL impairment group, BI ≤ 40 (*n* = 192)	
	Median	IQR (Q₁–Q₃)	Median	IQR (Q₁–Q₃)
Energy (S1)	3.33	2.67–4.00	2.00	1.25–3.00
Family roles (S2)	3.67	2.67–4.00	2.00	1.00–3.00
Language (S3)	4.20	4.00–5.00	3.00	2.00–4.00
Mobility (S4)	4.00	2.83–4.50	1.50	1.00–2.67
Mood (S5)	4.00	3.20–5.00	3.00	2.00–3.60
Personality (S6)	4.00	3.00–4.67	3.00	2.00–4.00
Self-care (S7)	4.00	3.20–4.80	1.70	1.00–2.85
Social Roles (S8)	3.40	2.40–4.00	1.60	1.00–2.65
Thinking (S9)	4.00	3.00–5.00	2.00	1.00–3.00
Upper extremity function (S10)	4.00	3.00–5.00	1.70	1.00–2.65
Vision (S11)	5.00	4.00–5.00	3.67	2.00–5.00
Productivity (S12)	3.00	2.00–4.00	1.00	1.00–2.00

IQR, interquartile range; Q₁, 25th percentile; Q₃, 75th percentile.

### Network structure in the mild–moderate group (BI > 40)

**Figure 1:**
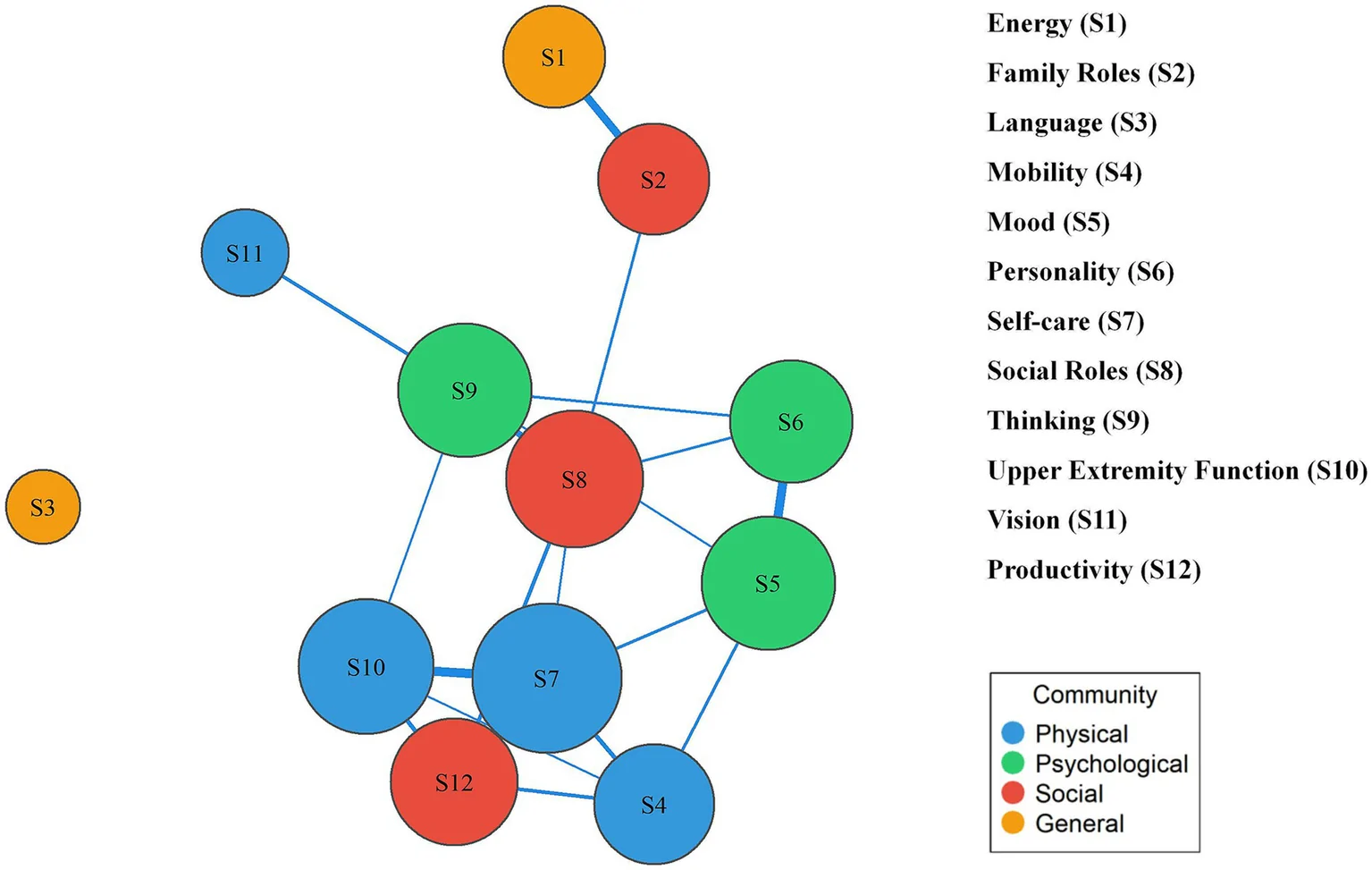
Estimated network structure of quality of life in the mild–moderate group (BI > 40, *n* = 259). Blue edges represent positive associations; red dashed edges represent negative associations. Edge thickness reflects the absolute strength of the association. Node size reflects strength centrality. Node colors indicate communities: blue = physical, green = psychological, red = social, orange = general.

In the Mild–Moderate Group (BI > 40), the network demonstrated good stability (CS = 0.595). Strength centrality was highest for Self-Care (S7; 1.209), followed by Social Roles (S8; 1.014) and Upper Extremity Function (S10; 0.978) (Table 3). The strongest edge was between SelfCare and Upper Extremity Function (0.506). Regarding bridge expected influence, Thinking (S9; 0.694) and Social Roles (S8; 0.650) showed the highest values. (Figure 1).

**Table 3:** Strength centrality and bridge expected influence (1-step) of quality-of-life domains by functional dependence group.

Dimension	Label	Mild–moderate ADL impairment group, BI > 40 (*n* = 259)		Severe ADL impairment group, BI ≤ 40 (*n* = 192)	
		Strength	Bridge EI	Strength	Bridge EI
Self-care	S7	**1.209**	0.465	0.894	0.210
Social roles	S8	**1.014**	**0.650**	0.917	0.620
Upper extremity	S10	**0.978**	0.362	**1.124**	0.436
Thinking	S9	0.952	**0.694**	**1.031**	**0.701**
Mood	S5	0.951	0.345	0.729	0.138
Productivity	S12	0.850	**0.632**	0.657	**0.657**
Mobility	S4	0.734	0.386	**1.012**	0.650
Personality	S6	0.779	0.157	0.757	0.188
Family roles	S2	0.592	0.446	0.648	0.351
Language	S3	0.000	0.000	0.722	**0.722**
Vision	S11	0.209	0.209	0.458	0.458
Energy	S1	0.446	0.446	0.351	0.351

Strength centrality = sum of absolute edge weights connected to a node.

Bridge expected influence (1-step) = sum of edge weights connecting a node to nodes in different communities.

Bold values (not shown) indicate top 3 in each column.

Betweenness and closeness are not reported due to instability (14).

Communities: physical (S4, S7, S10, S11), psychological (S5, S6, S9), social (S2, S8, S12), general (S1, S3).

### Network structure in the severe group (BI ≤ 40)

**Figure 2:**
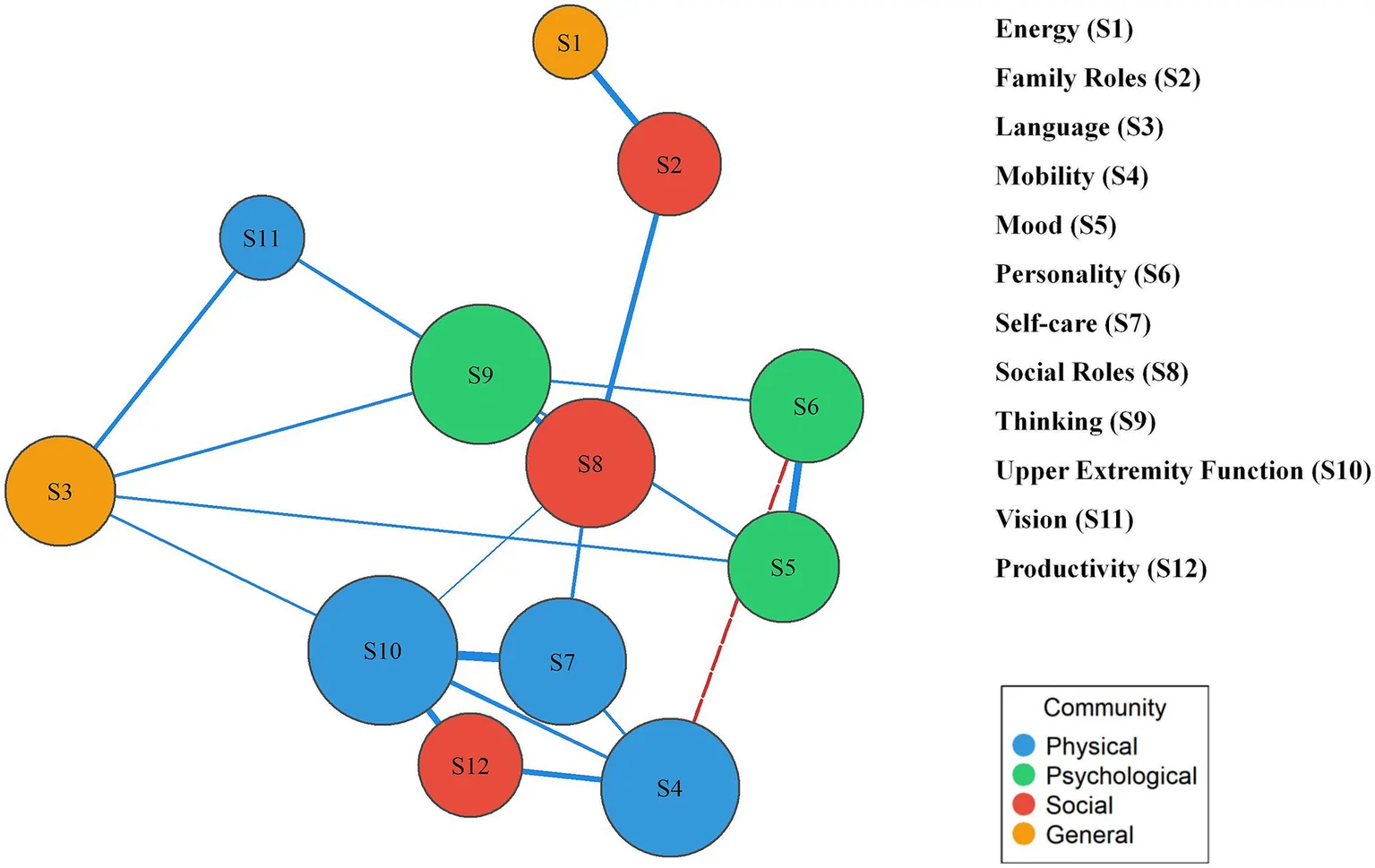
Estimated network structure of quality of life in the severe group (BI ≤ 40, *n* = 192). Blue edges represent positive associations; red dashed edges represent negative associations. Edge thickness reflects the absolute strength of the association. Node size reflects strength centrality. Node colors indicate communities: Blue = physical, green = Psychological, red = Social, orange = General.

In the Severe Group (BI ≤ 40), the network demonstrated acceptable stability (CS = 0.438). Strength centrality was highest for Upper Extremity Function (S10; 1.124), followed by Thinking (S9; 1.031) and Mobility (S4; 1.012) (Table 3). The strongest edge was again between SelfCare and Upper Extremity Function (0.506). Regarding bridge expected influence, Language (S3; 0.722) and Thinking (S9; 0.701) showed the highest values (Figure 2).

### Sensitivity analyses

The Network Comparison Test (NCT) showed that the severe group had significantly higher global strength (*p* = 0.021), although network structure did not differ between groups (*p* = 0.928); two edges showed significant between-group differences (Supplementary Table S1).

Additional sensitivity analyses using alternative correlation methods, community partitions, covariate adjustment, different BI cutoffs, and removal of overlapping domains all yielded generally consistent results, supporting the robustness of our findings (Supplementary Tables S2–S8).

## Discussion

This study applied network analysis to compare the interrelated structure of quality-of-life domains between stroke patients with mild-to-moderate (BI > 40) and severe (BI ≤40) functional impairment. In the mild-to-moderate group, the network was organized around both physical and social domains. In the severe group, physical domains (Upper Extremity Function, Mobility) remained central, but psychosocial-cognitive domains (Thinking, Social Roles) gained additional prominence. These findings indicate that strategies for improving quality of life may need to be tailored according to functional status, but physical function remains important across both levels of impairment. The Network Comparison Test (NCT) revealed that while global strength was significantly higher in the severe group (*p* = 0.021), suggesting a more densely connected network, the overall network structure did not differ between groups (*p* = 0.928) (Supplementary Table S1).

### Factors associated with quality of life in stroke survivors

Consistent with prior research, we observed significant associations between quality of life and several demographic and clinical factors (age, education level, monthly income, stroke type, and stroke severity) in our sample (17). In terms of age distribution, patients in the severe ADL impairment group showed a pronounced aging trend. Older stroke survivors are typically burdened with more complex comorbidities and reduced physiological reserve, which may limit their rehabilitation potential and exacerbate functional disability (17). In the economic dimension, 57.29% of the severe group had a monthly income below 3,000 CNY. The resource substitution theory posits that individuals with limited economic resources face greater difficulty in accessing alternative forms of support and compensation (9). Hemorrhagic strokes are often associated with more acute and severe neurological impairments, which aligns with our finding of significantly higher NIHSS scores in the severe group. The severity of neurological injury is related to the starting point for functional recovery, while the stroke type is often associated with the trajectory and potential of recovery (18).

### Mild–moderate group

The network analysis for patients with mild-to-moderate functional impairment (BI > 40) revealed a structure centered on physical and social domains. Self-Care (S7) showed the highest strength centrality (1.209), followed by Social Roles (S8; 1.014) and Upper Extremity Function (S10; 0.978) (Table 3). This indicates that perceived quality of life in this group is organized around both independent living and social participation. This finding aligns with the clinical reality that in the early or less severe stages of post-stroke recovery, regaining autonomy in activities of daily living is often the most immediate and salient goal for patients (19).

The strongest edge in the network, connecting Upper Extremity Function (S10) and Self-Care (S7) (weight = 0.506), highlights the functional dependency between these domains; the ability to use one’s arms and hands is a critical prerequisite for most self-care tasks (20).

Regarding bridge expected influence, Thinking (S9; 0.694) and Social Roles (S8; 0.650) showed the highest values. This suggests that even in mild-to-moderate impairment, cognitive function and social participation serve to integrate physical, emotional, and social aspects of quality of life (21).

Mood (S5) and Personality (S6) were strongly connected to each other, forming a distinct emotional cluster, while their connections to the core physical nodes (S7, S10) were relatively weaker. This structural pattern suggests that in patients with milder impairment, emotional well-being is relatively distinct from physical function. Language (S3) was isolated from the network (strength = 0.000). Vision (S11) and Energy (S1) also showed low centrality, suggesting they play a less central role in this group’s quality-of-life network (22).

### Severe group

In the severe group, the network showed high centrality for both physical domains (Upper Extremity Function, strength = 1.124; Mobility, 1.012) and psychosocial-cognitive domains (Thinking, 1.031; Social Roles, 0.917). This pattern is consistent with findings from a recent large-scale network analysis, which revealed that post-stroke cognitive impairments and activities of daily living form a densely interconnected network (23).

The strongest edge in the network, connecting SelfCare (S7) and Upper Extremity Function (S10) (weight = 0.506), remained identical to that in the mild–moderate group, highlighting the persistent functional dependency between upper extremity function and self-care ability across different levels of impairment.

Regarding bridge expected influence, Language (S3; 0.722) and Thinking (S9; 0.701) showed the highest values, followed by Social Roles (S8; 0.620). This indicates that communication and cognitive function serve as key connectors between different QOL domains in severe dependence. This structural pattern suggests a reorientation: when severe physical limitations become a permanent reality, the core endeavor of constructing quality of life shifts toward psychosocial adaptation—finding meaning and maintaining connections despite disability (24). This interpretation is supported by network analyses identifying cognitive-emotional appraisal as central in the adaptation process (23).

The heightened centrality of Social Roles (S8) suggests an intensified reliance on the social environment for support and identity validation (25). Collectively, these findings exemplify the broader principle that the architecture of quality of life exhibits different patterns across levels of functional status (21).

To further assess the robustness of our findings, we performed residualized network analyses controlling for age, NIHSS, stroke type, and income. The strength centrality rankings from the residualized networks were highly correlated with the original networks. This consistency indicates that our main findings are robust to potential confounding by these demographic and clinical variables.

A negative edge between Personality (S6) and Mobility (S4) was observed only in the severe group (weight = −0.188). Although personality has been linked to functional outcomes after stroke (26), this specific negative association was not statistically robust (95% CI crossed zero) and disappeared after covariate adjustment. Thus, it should be interpreted with caution.

## Conclusion and clinical implications

This study utilized network analysis to identify two distinct structural blueprints of health-related quality of life (HRQoL) in stroke survivors.

In patients with mild-to-moderate impairment, HRQoL is organized as a function-driven hierarchy centered on the synergistic recovery of Self-Care and Upper Extremity Function. Conversely, in those with severe dependence, quality of life appears as an adaptation-centric system where cognitive integrity and social connectedness become paramount.

These findings support a precision rehabilitation framework: prioritizing physical function synergies in milder cases and focusing on cognitive and emotional support in severe cases. Ultimately, the path to a sustainable life after stroke is not uniform but varies according to the architecture of the patient’s quality-of-life network.

### Limitations

Several limitations should be considered when interpreting the findings. First, the cross-sectional design precludes causal inferences and cannot capture the temporal dynamics of how these network structures emerge and evolve. Although time since stroke was well-balanced between groups (*p* = 0.056), with most participants in the acute phase, the cross-sectional design remains a limitation. Second, although our multi-center design enhanced sample diversity within the regional context, the generalizability of the identified network models requires validation across broader geographic regions and diverse healthcare systems. Third, aphasia status was not systematically recorded. Patients with severe aphasia who could not provide reliable self-report were excluded from the study. Thus, our findings may not generalize to stroke survivors with severe communication deficits. Fourth, the Barthel Index cutoff of 40 is clinically justified. Nevertheless, sensitivity analysis using an alternative cutoff (BI = 60) yielded consistent results (Supplementary Table S6; Supplementary Figure S1). Fifth, reliance on patient self-report for all quality-of-life domains, while essential for capturing subjective experience, may introduce common method variance. Across all sensitivity analyses (including alternative correlation methods, covariate adjustment, alternative community partitions, and removal of BI-overlapping domains) results remained generally consistent, supporting the robustness of our findings. Finally, future studies could employ latent profile analysis (LPA) to identify empirically derived subgroups of stroke survivors based on their multidimensional QOL profiles, complementing hypothesis-driven stratification based on clinical cutoffs. Prospective longitudinal network studies are also critical to trace the potential transition from a function-driven to an adaptation-centric model over the course of recovery.

## Data Availability

The original contributions presented in the study are included in the article/Supplementary material, further inquiries can be directed to the corresponding author.
