# Supplementary material for: The architecture of quality of life after stroke: a network comparison of mild-to-moderate versus severe functional impairment
Source: Front Neurol. 2026 Aug 19;17:1808859. doi: 10.3389/fneur.2026.1808859 (PMC13533719; doi:10.3389/fneur.2026.1808859)
Supplement: Supplementary file 1 [file Data_Sheet_1.docx]

Supplementary Table S1. Network Comparison Test (NCT) results between mild-moderate and severe groups

Supplementary Table S2. Sensitivity analysis comparing different correlation methods

Supplementary Table S3. Sensitivity analysis of bridge centrality with alternative community partition (3 communities)

Supplementary Table S4. Residualized network analysis controlling for age, NIHSS, stroke type, and income

Supplementary Table S5. Verification of negative edge in the severe group

Supplementary Table S6. Sensitivity analysis with alternative BI cutoff (BI = 60)

Supplementary Table S7. Node predictability (R²) for mild-moderate and severe groups

Supplementary Table S8. Sensitivity analysis removing BI-overlapping domains (SelfCare and Mobility)

| **Supplementary Table S1. Network. Comparison Test (NCT) results between mild-moderate and severe groups** | | | | | |
| --- | --- | --- | --- | --- | --- |
| **Test** | **Statistic** | **Value** | **95% CI** | **p-value** | **Interpretation** |
| Global Strength | Δ (difference) | 0.544 | [-0.12, 1.21] | 0.021 | Severe group had significantly higher global strength |
| Network Structure (M-test) | M | 0.151 | - | 0.928 | No significant difference in network structure |
| Individual edge tests | - | - | - | - | - |
| Mobility - Personality | Edge weight difference | 0.109 | - | 0.018 | Significant between-group difference |
| Family Roles - Vision | Edge weight difference | 0.119 | - | 0.006 | Significant between-group difference |
| *Note:* Global strength represents the sum of all absolute edge weights in the network. The M-test compares the overall network structure (invariance of edge weights). Individual edge tests were performed with 1,000 bootstrap iterations. CI for global strength difference was estimated via bootstrap. | | | | | |

| **Supplementary Table S2. Sensitivity analysis of correlation methods** | | | |
| --- | --- | --- | --- |
| **A. Mild-Moderate Group (n = 259)** | | | |
| Method | Top 3 Nodes (Strength) | Strength Values | Correlation with Polychoric |
| Polychoric (primary) | SelfCare, Social Roles, Upper Extremity | 1.209, 1.014, 0.978 | - |
| Spearman (sensitivity) | SelfCare, Upper Extremity, Social Roles | 1.118, 0.979, 0.924 | r = 0.956 |
| **B. Severe Group (n = 192)** | | | |
| Method | Top 3 Nodes (Strength) | Strength Values | Correlation with Polychoric |
| Polychoric (primary) | Upper Extremity, Thinking, Mobility | 1.124, 1.031, 1.012 | - |
| Spearman (sensitivity) | Thinking, Family Roles, Upper Extremity | 1.109, 1.020, 0.971 | r = 0.607 |
| Note: Polychoric correlation is recommended for ordinal Likert data and was used as the primary method. Spearman correlation was included for sensitivity. The lower correlation in the severe group (r = 0.607) reflects the restricted range and skewed distributions of QOL scores in severely impaired patients. Nevertheless, key nodes (SelfCare in mild-moderate; Thinking and Upper Extremity in severe) remained prominent across both methods. | | | |

| **Supplementary Table S3. Sensitivity analysis of bridge centrality** | | | | | |
| --- | --- | --- | --- | --- | --- |
| **A. Mild-Moderate Group (n = 259)** | | | | | |
| **Community Definition** | **Communities** | **Top 3 Nodes** | | **Bridge Strength** | **Correlation** |
| Original | Physical, Psychological, Social, General | Thinking, Social Roles, Productivity | | 0.694, 0.650, 0.632 | - |
| Alternative | Physical_General, Psychological, Social | Thinking, Social Roles, Productivity | | 0.694, 0.650, 0.632 | r = 1.000 |
| **B. Severe Group (n = 192)** | | | | | |
| **Community Definition** | **Communities** | **Top 3 Nodes** | | **Bridge Strength** | **Correlation** |
| Original | Physical, Psychological, Social, General | Language, Thinking, Productivity | | 0.722, 0.701, 0.657 | - |
| Alternative | Physical_General, Psychological, Social | Language, Thinking, Productivity | | 0.722, 0.701, 0.657 | r = 0.790 |
| *Note:* The alternative partition merged Physical and General into a single community (Physical_General), resulting in 3 communities. For the severe group, the correlation (r = 0.790) is primarily due to Mobility, which changed from a cross-community node (Physical → Social/Psychological) to an intra-community node (Physical_General), reducing its bridge strength from 0.650 to 0.505. Critically, Language, Thinking, and Productivity remained the top three bridge nodes across both partitions, supporting the robustness of our conclusions. | | | | | |
| **Table S4a. Comparison of strength centrality between original and residualized networks** | | | | | |
| **Group** | **Node** | **Original Strength** | **Residualized Strength** | | **Difference** |
| **Mild-Moderate (n=259)** | SelfCare | 1.209 | 1.235 | | 0.026 |
|  | Social Roles | 1.014 | 1.012 | | -0.002 |
|  | Upper Extremity | 0.978 | 0.865 | | -0.113 |
|  | Thinking | 0.952 | 0.961 | | 0.009 |
|  | Mood | 0.951 | 0.974 | | 0.023 |
|  | Productivity | 0.85 | - | | - |
|  | Personality | 0.779 | - | | - |
|  | Mobility | 0.734 | - | | - |
|  | Family Roles | 0.592 | - | | - |
|  | Energy | 0.446 | - | | - |
|  | Vision | 0.209 | - | | - |
|  | Language | 0.000 | - | | - |
| **Severe (n=192)** | Upper Extremity | 1.124 | 0.987 | | -0.137 |
|  | Thinking | 1.031 | 1.014 | | -0.017 |
|  | Mobility | 1.012 | - | | - |
|  | Social Roles | 0.917 | 0.785 | | -0.132 |
|  | SelfCare | 0.894 | 0.914 | | 0.02 |
|  | Personality | 0.757 | - | | - |
|  | Mood | 0.729 | 0.734 | | 0.005 |
|  | Language | 0.722 | - | | - |
|  | Productivity | 0.657 | 0.657 | | 0.000 |
|  | Family Roles | 0.648 | - | | - |
|  | Vision | 0.458 | - | | - |
|  | Energy | 0.351 | - | | - |
| Note: Only nodes with changes > 0.01 are shown for residualized networks. Full results available upon request. Dashes indicate nodes that were not in the top 5 of residualized networks. | | | | | |
|  |  |  |  | |  |
| **Table S4b. Summary of residualized network analysis** | | | | | |
| **Group** | **Correlation (Original vs Residualized)** | **Top 3 Original** | **Top 3 Residualized** | | **Agreement** |
| Mild-Moderate | r = 0.886 | SelfCare, Social Roles, Upper Extremity | SelfCare, Social Roles, Mood | | 2/3 match |
| Severe | r = 0.879 | Upper Extremity, Thinking, Mobility | Thinking, Upper Extremity, SelfCare | | 2/3 match |
| Note: Residualized networks were estimated after regressing out age, NIHSS, stroke type, and income from each QOL domain. The high correlations (r > 0.85) and substantial overlap in top nodes indicate that our findings are robust to these potential confounders. | | | | | |

| **Supplementary Table S5. Verification of negative edge in the severe group** | | | |
| --- | --- | --- | --- |
| **A. Bootstrap analysis (200 resamples)** | | | |
| Edge | Original Weight | Bootstrap Mean (SD) | 95% CI |
| Personality — Mobility | -0.188 | -0.121 (0.102) | [-0.286, 0] |
| **B. Residualized network (controlling for age, NIHSS, stroke type, income)** | | | |
| Network | Weight of Personality — Mobility | Presence |  |
| Original network | -0.188 | Present |  |
| Residualized network | -0.009 | Absent |  |
| *Note:* The 95% confidence interval crosses zero, indicating that this negative association did not reach statistical significance. The bootstrap p-value is not reported due to boundary effects (CI upper bound = 0). | | | |

| **Supplementary Table S6. Sensitivity analysis with alternative BI cutoff (BI = 60)** | | | | | |
| --- | --- | --- | --- | --- | --- |
| **Group** | **Primary (BI = 40)** | | **Alternative (BI = 60)** | | **Correlation (r)** |
|  | Sample | Top 3 Strength | Sample | Top 3 Strength | r |
| Mild-Moderate | n=259 | SelfCare (1.209), Social Roles (1.014), Upper Extremity (0.978) | n=398 | SelfCare (1.299), Thinking (0.957), Upper Extremity (0.924) | 0.886 |
| Severe | n=192 | Upper Extremity (1.124), Thinking (1.031), Mobility (1.012) | n=53 | SelfCare (1.028), Thinking (1.026), Upper Extremity (0.977) | 0.879 |
| *Note:*Using BI = 60 resulted in a smaller severe group (n = 53) but yielded highly consistent centrality rankings (r > 0.85), supporting the robustness of our findings. | | | | | |

| **Supplementary Table S7. Node predictability (R²) for mild-moderate and severe groups** | | | |
| --- | --- | --- | --- |
| **Label** | **Dimension** | **Mild-Moderate R²** | **Severe R²** |
| S7 | Self-care | 0.725 | 0.759 |
| S10 | Upper Extremity | 0.686 | 0.778 |
| S12 | Productivity | 0.591 | 0.648 |
| S4 | Mobility | 0.561 | 0.618 |
| S5 | Mood | 0.541 | 0.591 |
| S8 | Social Roles | 0.522 | 0.645 |
| S9 | Thinking | 0.519 | 0.655 |
| S6 | Personality | 0.462 | 0.512 |
| S2 | Family Roles | 0.418 | 0.442 |
| S1 | Energy | 0.345 | 0.299 |
| S11 | Vision | 0.209 | 0.434 |
| S3 | Language | 0.000 | 0.522 |
| Node predictability (R²) values ranged from 0.000 to 0.725 in the mild-moderate group and from 0.299 to 0.778 in the severe group. SelfCare and Upper Extremity Function showed the highest predictability in both groups (R² > 0.68), indicating that these domains are strongly embedded within the HRQoL network and are well-explained by other QOL dimensions. | | | |

| **Supplementary Table S8. Sensitivity analysis removing BI-overlapping domains (SelfCare and Mobility)** | | | |
| --- | --- | --- | --- |
| **Group** | **Analysis** | **Top 5 Strength Nodes (values)** | **Correlation with Original** |
| **Mild-Moderate** | Original (12 domains) | S7 (1.209), S8 (1.014), S10 (0.978), S9 (0.952), S5 (0.951) | - |
|  | After removing S7, S4 | S8 (0.960), S10 (0.832), S12 (0.814), S5 (0.807), S6 (0.792) | r = 0.927 |
| **Severe** | Original (12 domains) | S10 (1.124), S9 (1.031), S4 (1.012), S8 (0.917), S7 (0.894) | - |
|  | After removing S7, S4 | S9 (1.022), S8 (0.949), S10 (0.839), S5 (0.747), S12 (0.717) | r = 0.881 |
| *Note:*S4 = Mobility, S5 = Mood, S6 = Personality, S7 = SelfCare, S8 = Social Roles, S9 = Thinking, S10 = Upper Extremity Function, S12 = Productivity. The high correlations (r > 0.88) indicate that removing domains most conceptually overlapping with the Barthel Index did not substantially alter the network structure, supporting the robustness of our findings against potential circularity. | | | |
